# Genetics of Calcific Aortic Stenosis: A Systematic Review

**DOI:** 10.3390/genes15101309

**Published:** 2024-10-10

**Authors:** Vassilios S. Vassiliou, Nicholas Johnson, Kenneth Langlands, Vasiliki Tsampasian

**Affiliations:** 1 Norwich Medical School, University of East Anglia, Norwich NR4 7TJ, UK; nicholas.johnson@uea.ac.uk (N.J.); tsampasian@doctors.org.uk (V.T.); 2 Fitzwilliam College, University of Cambridge, Cambridge CB3 0DG, UK; 3 Institute of Continuing Education, University of Cambridge, Cambridge CB23 8AQ, UK; kenneth.langlands@ice.cam.ac.uk

**Keywords:** aortic stenosis, calcific

## Abstract

**Background:** Calcific aortic stenosis is the most prevalent valvular abnormality in the Western world. Factors commonly associated with calcific aortic stenosis include advanced age, male sex, hypertension, diabetes and impaired renal function. This review synthesises the existing literature on genetic associations with calcific aortic stenosis. **Methods:** A systematic search was conducted in the PubMed, Ovid and Cochrane libraries from inception to 21 July 2024 to identify human studies investigating the genetic factors involved in calcific aortic stenosis. From an initial pool of 1392 articles, 78 were selected for full-text review and 31 were included in the final qualitative synthesis. The risk of bias in these studies was assessed using the Newcastle Ottawa Scale. **Results:** Multiple genes have been associated with calcific aortic stenosis. These genes are involved in different biological pathways, including the lipid metabolism pathway (*PLA*, *LDL*, *APO*, *PCSK9*, *Lp-PLA2*, *PONS1*), the inflammatory pathway (*IL-6*, *IL-10*), the calcification pathway (*PALMD*, *TEX41*) and the endocrine pathway (*PTH*, *VIT D*, *RUNX2*, *CACNA1C*, *ALPL*). Additional genes such as *NOTCH1*, *NAV1* and *FADS1/2* influence different pathways. Mechanistically, these genes may promote a pro-inflammatory and pro-calcific environment in the aortic valve itself, leading to increased osteoblastic activity and subsequent calcific degeneration of the valve. **Conclusions:** Numerous genetic associations contribute to calcific aortic stenosis. Recognition of these associations can enhance risk stratification for individuals and their first-degree relatives, facilitate family screening, and importantly, pave the way for targeted therapeutic interventions focusing on the identified genetic factors. Understanding these genetic factors can also lead to gene therapy to prevent calcific aortic stenosis in the future.

## 1. Introduction

The aortic valve serves as the primary outlet valve of the heart, operating approximately 40 million times annually to maintain a one-way flow of blood from the left ventricle (LV), the main pumping chamber of the heart, to the aorta, the principal artery responsible for distributing blood throughout the body. Anatomically, the aortic valve consists of three distinct layers [1]. The *Fibrosa* is the outermost layer; it is rich in collagen, located closest to the ascending aorta and is frequently susceptible to disease. Secondly, the *Ventricularis* is positioned adjacent to the LV and is rich in elastin. This layer is relatively shielded from disease. Thirdly, the *Spongiosa* is situated between the fibrosa and ventricularis and is predominantly composed of proteoglycans.

**Figure 1:**
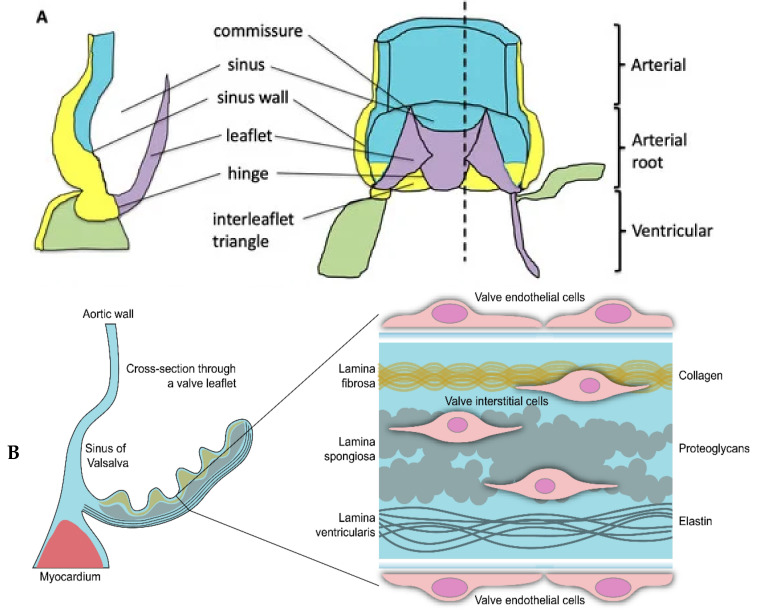
A simplified illustration of the human aortic valve is shown. Panel (**A**) depicts the anatomical structure of the aortic valve in relation to aorta and ventricle. Reproduced with permission from Henderson et al. [2] under a creative commons attribution 4.0 international license. Panel (**B**) focuses on the aortic valve leaflet. The left side presents a schematic cross-section of the non-coronary leaflet of the aortic valve. The enlarged section on the right highlights the three-layered structure of the extracellular matrix, indicating the locations of the aortic valve endothelial cells (VECs) and valvular interstitial cells (VICs). Reproduced with permission from Rutkovskiy et al. [1] under a creative commons attribution 4.0 international license.

In healthy aortic valves, valvular interstitial cells (VICs) are distributed across all layers, ensuring the structural integrity of the valve (Figure 1).

In disease, however, VICs undergo myofibrogenic and osteogenic differentiation, leading to extracellular matrix (ECM) expansion, collagen accumulation and formation of calcific nodules [3].

This process results in thickening and stiffening of the valve leaflets, which over time causes narrowing of the valve opening, a condition known as aortic stenosis. When this narrowing is due to valve calcification, it is referred to as calcific aortic stenosis (CAS), which impedes the smooth flow of blood from the LV to the aorta.

CAS is the most prevalent form of aortic stenosis and the most common valvular abnormality in the Western world [4], affecting approximately 13 million people globally [5]. Primarily an age-related disease, its prevalence is anticipated to double in the next 20 years with the ageing population [6]. CAS is multifactorial, involving environmental, mechanical and genetic factors, with recent research increasingly emphasising the genetic basis of the disease [7].

If the pressure overload caused by CAS is not relieved, it leads to heart failure and premature death. Without treatment, it is estimated that 50% of patients with severe symptomatic CAS will die within two years [8]. Currently, there are no pharmacological treatments available to slow, reverse or prevent CAS. Consequently, the primary treatment involves interventions to relieve the mechanical obstruction and pressure overload [9], typically through open-heart surgery to replace the calcified valve with a mechanical one, or when the anatomy is favourable, the less invasive transcatheter aortic valve implantation (TAVI) is an option.

While certain risk factors such as diabetes, hypertension and renal failure are associated with incidence of CAS [7], there is growing interest in the genetic aspects of the disease. Although individual studies have reported genetic associations with CAS, no systematic review has yet synthesised all the studies on this topic.

Understanding the genetic components of CAS provides valuable insights into the molecular mechanisms driving the disease and can help identify potential therapeutic targets. Additionally, this knowledge may improve early detection of individuals at increased risk, explain the varying incidence rates among different populations (for example, White populations have a three-fold higher risk of CAS compared to Black populations [10]), support the development and use of polygenic risk scores [11,12], as well as guiding screening and counselling efforts.

The aim, therefore, was to conduct a systematic review of the published literature, and to synthesise and critically appraise the data.

## 2. Materials and Methods

A systematic review was carried out in accordance with the PRISMA guidelines. The Pubmed, Ovid and Cochrane databases were searched from inception to 21 July 2024, using the following terms: 

*(calcific aortic stenosis) AND ((gene) OR (genome) OR (loci) or (variant) OR (mutation) OR (genetics)) and corresponding MeSH (Medical Subjects Heading) terms*.

The search results were imported into the dedicated systematic review software, Covidence [13], for screening. Studies were included in the systematic review if they investigated specific genes associated with CAS. Eligible studies included any original human research (observational, prospective, retrospective or randomised) involving adults (>18 years old) that provided a genetic association with CAS. Editorials, reviews, abstracts, case reports and animal studies were excluded. Studies solely focusing on aortic calcification without valve narrowing (i.e., without stenosis) or only on the genetic influence in the progression of aortic stenosis without data on incident CAS were also excluded. Additionally, studies involving patients with bicuspid aortic valves (i.e., non-CAS) were excluded, unless CAS data were provided.

After removing duplicates, each title and abstract was screened by two individuals. Full articles were reviewed and included if they documented any association with the incidence of CAS. Disagreements were resolved through discussion and adjudication by a third individual. The Newcastle–Ottawa Scale for non-randomised studies [14] was utilised to assess quality and risk of bias.

**Figure 2:**
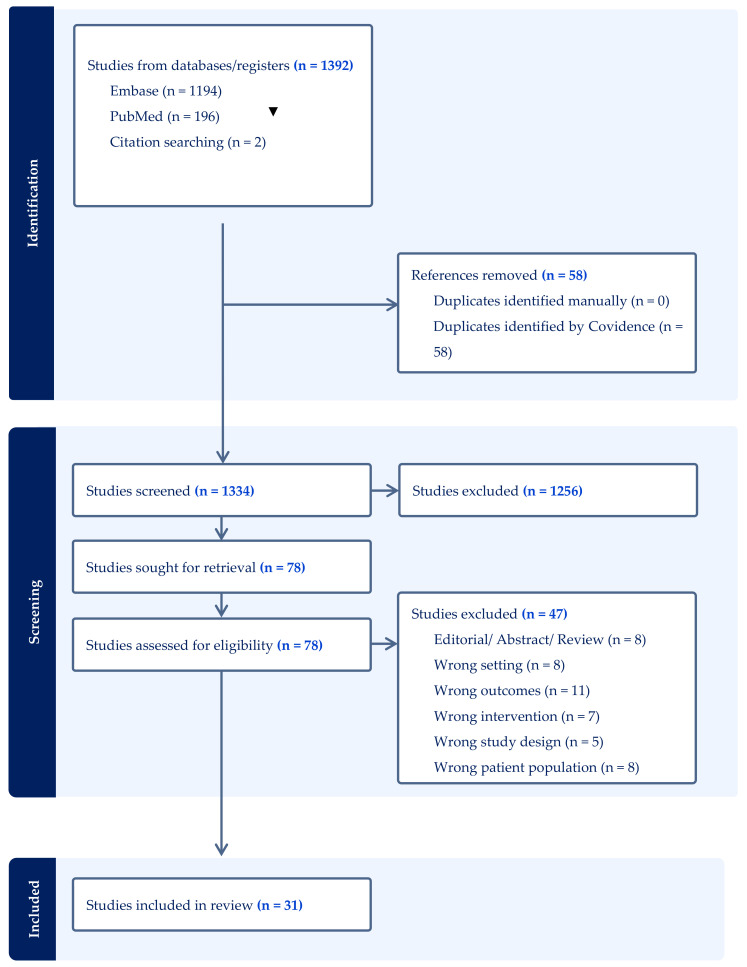
PRISMA flow diagram summarising study selection.

The study was registered with PROSPERO (registration number CRD42024563615) and reported according to Preferred Reporting Items for Systematic Reviews and Meta-Analyses (PRISMA) guidelines, as illustrated in Figure 2 and Supplementary Table S1.

## 3. Results

Out of 1392 articles initially identified in the databases, 58 duplicates were removed, leaving 1334 articles for title and abstract review. This process resulted in 78 articles undergoing full-text review, of which 31 articles were included in this systematic review. The papers were collectively organised in pathways based on genes investigated (see graphical abstract), along with descriptions of potential mechanistic effects, and are shown in Table 1. The studies were all of good quality (see Supplemental Table S2 for Newcastle–Ottawa scale results).

In addition, studies that reported on multiple variants or multiple cohorts are also shown for ease of reference in Supplementary Tables S3 and S4. 

### 3.1. Lipid Metabolism Genes and CAS 

#### 3.1.1. Lipoprotein (A)

**Figure 3:**
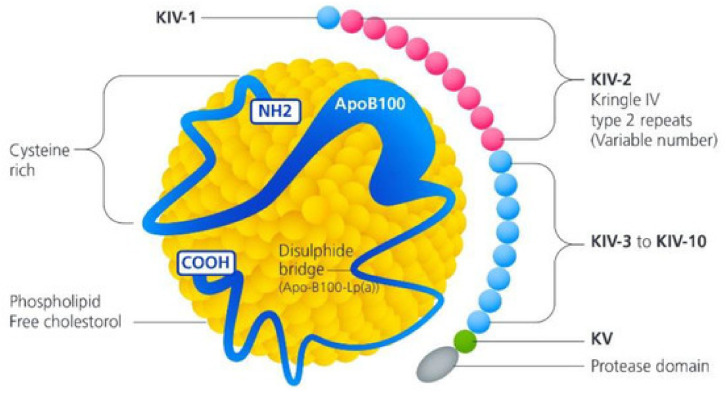
Schematic representation of Lp(a). Lp(a) consists of apolipoprotein B100 covalently linked to apo(a), and high levels enable a pro-inflammatory and pro-calcific environment. Variants with smaller kringle IV type 2 repeats are associated with higher blood Lp(a) levels; hence, the plasma concentration of Lp(a) is genetically determined. Reproduced with permission from Telyuk et al. [46] under a creative commons attribution 4.0 international license.

One of the most studied genes in relation to CAS is the lipoprotein (A) (*LPA*) gene, located on chromosome 6 at position 6q25.3-26. The *LPA* gene encodes apoliporotein(a) [apo(a)], a protein that contains a series of kringle domains with triple loop structures stabilised by three internal disulphide bonds [44]. This structure makes apo(a) comparable to plasminogen, a glycoprotein involved in fibrinolysis. As a result, apo(a) inhibits fibrinolysis by competitively preventing plasminogen from binding to fibrin. Additionally, apo(a) is a major component of lipoprotein(a) [Lp(a)], a cholesterol-rich particle that includes a molecule of apolipoprotein B100 covalently linked to a molecule of apo(a) [45], as shown in Figure 3. Thus, Lp(a) levels are mostly genetically determined by a variation in kringle IV type 2 repeat numbers at the LPA apo(a) encoding gene [45].

Traditionally, elevated plasma Lp(a) is linked to an increased risk of cardiovascular events such as myocardial infarctions and strokes, independently of other lipids like low density lipoprotein (LDL). In 2013, Thanassoulis et al. [15] however, demonstrated through three cohort studies (Framingham Heart Study (FHS) cohort [47], the Age, Gene/Environment Susceptibility–Reykjavik Study (AGES-RS) cohort [48], and white European participants in the Multi-Ethnic Study of Atherosclerosis (MESA) [49]) that one single nucleotide polymorphism (SNP) on chromosome 6, rs10455872, located within intron 25 of the *LPA* gene, achieved genome-wide significance for aortic valve calcification after adjustment for age and sex (odd ratio [OR] = 2.05; 95% confidence interval [CI] = 1.63–2.57, *p* = 9.0 × 10^−10^). This meant that the presence of the G allele at this location more than doubled the risk of aortic valve calcification. This finding was further validated in the Malmö Diet and Cancer Study (MDCS) [50], which included 28,193 individuals followed for 14 years, during which 308 (1.1%) developed CAS. After adjusting for other risk factors, notably male sex, increasing age, smoking and body mass index (BMI), the rs10455872 SNP remained independently associated with CAS (hazard ratio [HR] = 1.68, CI = 1.32–2.15; *p* = 3 × 10^−5^). Subsequent studies, including large cohorts from the UK Biobank (2574 CAS cases, 405,829 controls) [19], Million Veterans Program (14,451 CAS cases, 398,544 controls) [12] and the EPIC-Norfolk Study (508 cases, 20,421 controls) [23] also confirmed the association between *LPA* and CAS.

High Lp(a) levels have been shown to contribute to a pro-inflammatory and pro-calcific environment in the aortic valve [51], and various mechanisms have been proposed for how this leads to valve calcification. One mechanism suggests that after being transferred from the bloodstream into the inner wall of the aortic valve cusps [52], Lp(a) induces cholesterol deposition similar to LDL cholesterol, leading to thickening of the valve cusps. This is supported by the structural similarity between Lp(a) and LDL. Another potential mechanism involves Lp(a) promoting thrombosis by competing with plasminogen and inhibiting plasmin from dissolving fibrous clots, which could result in fibrin deposition and aortic valve calcification [53]. Furthermore, within the valve layers, oxidised phospholipids are converted by the enzyme Lp-phospholipase-2 into lysophosphatidylcholine, promoting valve mineralisation. This is subsequently converted into lysophosphatidic acid by the enzyme autotaxin on Lp(a), stimulating VICs to produce osteoblastic transcription factors [54,55]. Finally, oxidised phospholipids elicit an inflammatory response with macrophages, T-lymphocytes and mast cells producing microcalcifications within the endothelium [56]. Overall, these processes promote a pro-inflammatory and pro-fibrotic environment that leads to calcific degeneration of the aortic valve cusps, resulting in CAS.

The significance of these studies lies in their support for the genetic effect of *LPA* in determining blood concentrations of Lp(a) and its association with CAS. Consequently, reducing blood Lp(a) levels could represent a novel pharmacotherapeutic approach to preventing CAS. Previous methods to reduce Lp(a), such as nicotinic acid and plasmapheresis, were either poorly tolerated or invasive. However, newer pharmacotherapies have overcome these limitations. Proprotein Convertase Subtilisin/Kexin 9 (PCSK9) inhibitors, available as twice-monthly injections, are generally well-tolerated. Clinical trials assessing PCSK9 inhibitors primarily for LDL reduction have also evaluated their effects on Lp(a) and CAS.

The FOURIER study, which randomised 27,564 patients to PCSK9 inhibitor (evolocumab) or placebo, showed a 60% decrease in LDL and a 26.9% reduction in Lp(a) [57]. A FOURIER sub-study indicated a non-statistical decrease in CAS in the evolocumab group after only two years OR = 0.6 (CI = 0.40–1.09) [58], meaning that there could be potentially a reduction in CAS of 34%. Nonetheless, this result is quite reassuring, opening the way to prospective trials with longer treatment duration in this area.

Two ongoing randomised controlled trials (RCTs) are further exploring this hypothesis. One trial is randomising 160 patients [59] with CAS to receive either a PCSK9 inhibitor or a statin, and another is randomising 140 patients [60] to receive either a PCSK9 inhibitor or placebo, aiming to determine whether PCSK9 inhibitors can delay the progression of CAS.

Additionally, a different class of medication, inclisiran (a double-stranded small interfering RNA that suppresses PCSK9 in the liver), has also shown a 20% reduction in Lp(a). Inclisiran’s significant advantage over PCSK9 inhibitors is its biannual injection schedule compared to biweekly injections.

Current studies on inclisiran [61] have reported reductions in LDL and Lp(a), but cardiovascular outcomes, including the incidence of CAS, are still awaited.

#### 3.1.2. Low Density Lipoprotein

Continuing with the lipid theme, a study by Smith et al. [62] utilised the Malmö study and a genetic risk score for LDL, based on 57 SNPs, to investigate the incidence of CAS. Within this cohort of 28,461 patients, there were 473 cases of CAS. The LDL genetic risk score demonstrated an HR = 1.28 (CI = 1.04–1.57; *p* = 0.02), even after adjusting for age, sex, height, weight, diabetes, hypertension and smoking. This finding was further confirmed by Allara et al. [25], who performed polygenic LDL estimation and assessed its association with multiple endpoints, including aortic stenosis. Their study showed a significant association with CAS (OR per one standard deviation [SD] increase, 1.46 [95% CI = 1.25–1.70; *p* < 0.003]). This means that for every increase of one SD in LDL, the associated increase in CAS was 46%. These results provide strong evidence that higher LDL levels are associated with a higher incidence of CAS.

This observation could potentially revolutionise the approach to pharmacotherapy in CAS. It might explain why multiple studies, including those using statins (such as SALTIRE [63], TASS [64], SEAS [65] and ASTRONOMER [66]) have failed to identify a medication that effectively reduces the progression of calcification in aortic stenosis. There are two possible explanations for this: first, the overall effect of LDL in CAS may be small, with other factors such as Lp(a) having a more significant impact; second, LDL might initiate CAS and sustain its early progression, but after a certain threshold of calcification (as seen, for example, in mild aortic stenosis), the progression to moderate and severe aortic stenosis may become independent of LDL. Consequently, offering LDL-reducing therapy for moderate or severe aortic stenosis might be too late; it may need to be provided at the earliest stage of aortic calcification.

Moreover, this suggests that targeting a single factor may be insufficient. For instance, reducing LDL without addressing Lp(a) may not achieve the desired effect. This genetic understanding identifies areas for improvement in research and suggests that future studies should consider early pharmacotherapy for CAS and ensure that all possible mechanisms identified through genetic associations are targeted.

### 3.2. PCSK9-Proprotein Convertase Subtilisin/Kexin 9

PCSK9 is located on chromosome 1 at position 1p32.3 and plays a crucial role in cholesterol metabolism by regulating LDL receptor levels, thereby influencing the removal of LDL particles from the plasma. A gain of function in the *PCSK9* gene reduces LDL receptors, allowing LDL to remain in the plasma longer, which is associated with adverse cardiovascular events [67].

**Figure 4:**
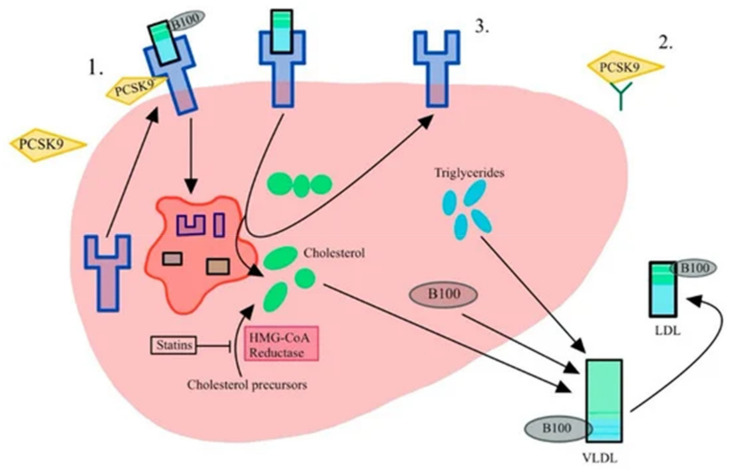
Mechanism of action of PCSK9 inhibitors. PCSK9 identifies and marks the LDL receptor for phagocytosis (number 1), thereby allowing more LDL to circulate in the bloodstream. PCSK9 inhibitors bind to LDL receptors (2), and thus allow the LDL receptors to clear more LDL particles (shown in 3). Reproduced from Beltran et al. [68] under a creative commons attribution 4.0 international license.

Conversely, a loss of function leads to more LDL receptors, which remove LDL more effectively, resulting in lower LDL levels in the bloodstream and, subsequently, a reduced cardiovascular risk profile. Figure 4 shows the effect of PCSK9 inhibition, an effect analogous to a loss of function mutation.

In a 2016 study, Langsted et al. [26] investigated the loss of function in the *PCSK9* R46L region using data from the Copenhagen General Population Registry, which included 1463 patients with CAS and 100,503 controls. Among the participants, 2606 were mutation carriers, with 26 events (1.0%), compared to 1437 events (1.4%) among 100,477 non-carriers. The OR for CAS in carriers versus non-carriers was 0.64 (95% CI, 0.44–0.95), after adjusting for age, sex, LDL and Lp(a) levels. Similar results were also obtained by Perrot et al. [27], who identified an OR = 0.80 (CI = 0.70–0.91, *p =* −0.001) for the same variant.

Several mechanisms likely contribute to the reduction in CAS with *PCSK9* loss of function. Firstly, its direct effect on lowering LDL and Lp(a) levels can diminish the pro-inflammatory and pro-calcific environment in the valve. Additionally, higher levels of PCSK9 are known to trigger the expression of inflammatory cytokines, adhesion molecules, and chemoattractants, leading to increased monocyte adhesion and inflammation [69]. PCSK9 levels were found to be elevated in explanted calcific aortic valve VICs, while PCSK9 inhibition in these valves reduced VIC expression by more than 50%, suggesting that PCSK9 has an independent effect beyond LDL and Lp(a).

Like the *LPA* gene, the above evidence suggests that inhibiting PCSK9 could potentially prevent or slow the progression of CAS.

### 3.3. Apolipoprotein

#### 3.3.1. Apolipoprotein E

Another well-studied gene affecting lipid metabolism is apolipoprotein E *(APOE),* located on chromosome 19 at position 19q13. This gene encodes APOE, a crucial component of plasma lipoproteins including chylomicrons, very low-density lipoproteins (VLDL) and high-density lipoproteins (HDL). APOE mediates the binding of lipids to specific receptors in liver cells, facilitating the clearance of triglyceride-rich lipoproteins from the bloodstream. *APOE* has three alleles, ε2, ε3 and ε4, which result in different isoforms of the APOE protein (APOE 2/2, 2/3, 2/4, 3/3, 3/4, 4/4). The 3/3 is most common, accounting for 60% of the population in the US [41]. These isoforms vary slightly in their amino acid sequence, affecting their function and association with various diseases. The ε3 allele is considered neutral, while the ε2 allele appears to protect against cardiovascular diseases, and the ε4 allele is linked to a higher risk for cardiovascular diseases and Alzheimer’s dementia.

In 2002, Avakian et al. [28] investigated the potential role of apolipoproteins A1, B and E in CAS. In a case–control study, they compared the frequency of these genes in 62 non-diabetic patients with CAS and controls matched for age, sex, BMI and hypertension. The only gene that showed a significant difference in distribution between the CAS patients and controls was *APOE* ε2. Among CAS patients, 32% had ε2 genotypes compared to 14% in controls (*p* = 0.007), leading the authors to conclude that the ε2 genotype might confer susceptibility to CAS independently of serum lipoprotein levels. This finding was further supported in 2003 by Novaro et al. [29], who investigated 43 CAS patients and 759 controls. In multivariable analysis, the *APOE4* genotype (APOE 2/4 and 3/4) was independently associated with CAS, even when adjusted for sex, age, presence of CAD and LDL levels. However, in contrast to these studies, Ortlepp et al. [30] found no association between *APOE* ε2, ε3 or ε4 genotypes and CAS in a study of 538 CAS patients with 536 controls.

In 2017, Kritharides et al. [32] further investigated this potential association using data from 46,615 individuals from the Copenhagen General Population Study. During a follow-up period of 37 years, 345 individuals developed CAS. The risk associated with four subgroups, based on the presence or absence of ε2 and Lp(a) levels, is shown in Table 2.

Since the only significant association was observed in non-carriers with high Lp(a), the authors concluded that while *APOE* is associated with CAS, this relationship is likely mediated through its effects on reducing Lp(a) levels rather than having a direct causative effect.

#### 3.3.2. Apolipoprotein B 

Gaudreault et al. [31] further compared SNPs from 457 patients with severe aortic stenosis who underwent surgical aortic valve replacement to 3294 controls. The *APOB* gene, which encodes apolipoprotein B (a primary component of LDL), was the focus. They identified a significant association between a missense mutation in the *APOB* gene (rs1042031, E4181K) and aortic stenosis (*p* = 0.00001). Additionally, a second SNP located 5.6 kilobases downstream of the *APOB* stop codon (rs6725189) was also linked to the disease (*p* = 0.000013). Although the study did not specify that all patients had CAS, the average age of 70 at the time of surgery suggests that most had CAS rather than bicuspid aortic valve disease, which typically requires surgery by age 60.

Furthermore, Wang et al. [33] genotyped two SNPs in the *APOB* gene (rs6725189 and rs693) in 314 Chinese patients with CAS and 652 age- and sex-matched controls. They found that the presence of the T allele (TT/CT vs. CC) in rs693 was associated with CAS (OR = 1.58, CI 1.18–2.10; *p* = 0.002) and that the GT genotype in rs6725189 was also associated with CAS (OR = 1.82, CI 1.14–2.93; *p* = 0.013).

In summary, the association between *APOE* and CAS appears strong, with some authors suggesting this relationship is mediated through *APOE’s* effect on Lp(a) [70]. This reinforces the importance of Lp(a), and studies designed to investigate CAS reduction when Lp(a) is lowered will help address this knowledge gap. The potential association of *APOB* with CAS remains unclear.

### 3.4. Lipoprotein-Associated Phospholipase A₂

Elevated levels of lipoprotein-associated phospholipase A_2_ (*Lp-PLA2*) have been associated with CAS. In 2020, Perrot et al. [34], investigated four SNPs at the *PLA2G7* locus associated with activity or mass of Lp-PLA2: rs7756935, rs1421368, rs1805017 and rs4498351. Genetic associations were pooled from eight studies (QUEBEC-CAVS, UK Biobank [71], EPIC Norfolk [72], GERA [34], CAVS France 1, 2 and 3 [53] and MDCS [50]) totalling 10,137 cases and 434,585 controls. None of these SNPs were associated with CAS, suggesting that *Lp-PLA2* was unlikely to be a causal risk factor or therapeutic target.

### 3.5. Paraoxonase 1

Paraoxonase 1 (*PONS1*) is involved in the hydrolysis of HDL particles, and the beneficial effects of HDL in cardiovascular disease is partly attributed to *PONS1* [73]. Moura et al. [35] studied 67 patients with CAS and 251 controls, investigating two SNPs of *PON1*: rs662 and rs854580. For rs662, there was a significant association with the R allele in CAS patients (*p* = 0.01), whereas no association was found with rs854580. While this association requires further evaluation, it suggests that HDL metabolism may play a role in the development of CAS.

### 3.6. Inflammatory Pathway Genes and CAS 

#### 3.6.1. Interleukin 6

Interleukin 6 (IL-6) is located on chromosome 7 at position 7p15.3. It is a pro-inflammatory cytokine involved in inflammatory and immune responses. It has been suggested that the increased inflammatory response associated with the *IL-6* gene facilitates valve damage and calcification [74]. Specifically, the IL-6 protein as dictated by the *IL-6* gene, can activate VICs and induce their transformation into osteoblast-like cells, contributing to calcium deposition in the valve matrix. Furthermore, elevated IL-6 levels can lead to an imbalance between matrix synthesis and degradation, promoting calcification and fibrosis in the aortic valve. 

Theriault et al. [19] first investigated the effect of *IL-6* variants in four cohorts totalling 5115 CAS patients and 354,072 controls. For CAS, the *IL-6* rs2069832-A allele had an OR = 1.27 (CI = 1.19–1.36; *p* = 6.71 × 10^−10^). This finding was further supported by Junco-Vicente et al. [36,59], who studied the *IL*-6 rs1800795 allele in 175 CAS patients and 478 controls. The presence of the rs1800795-C allele was associated with an increased risk of CAS (OR = 1.72, CI 1.19–2.52; *p* = 0.005). 

Another significant study by Small et al. in 2023 reported on the Million Veteran Program [12]. They investigated multiple SNPs in 14,451 CAS patients and 398,544 controls, with *IL-6* rs1474347 confirming a positive association for CAS (OR = 1.10, CI = 1.07–1.13; *p* = 6.7 × 10^–14^).

#### 3.6.2. Interleukin 10

Another gene associated with inflammation is interleukin 10 (*IL-10*), located on chromosome 1 at position 1q31-32. Unlike IL-6, IL-10 has an anti-inflammatory role by suppressing pro-inflammatory cytokines such as IL-1 and tumour necrosis factor α (TNF-α), which promote inflammation and calcification of heart valves. Additionally, IL-10 has been shown to reduce oxidative stress, thereby decreasing the differentiation of valvular interstitial cells (VICs) into osteoblast-like cells that deposit calcium in the valve [75]. 

Gaudreault et al. [31] investigated 457 patients with CAS and compared them to a publicly available group of 3294 controls. In their study, they examined 52 SNPs across 7 genes, including *IL*-10. Six of these SNPs (rs1800896, rs1800872, rs1554286, rs3024491, rs3021094, rs1554286) had a significantly higher frequency in CAS cases than in controls (*p*-values ranging from 2.97 × 10^−4^ to 6.21 × 10^−11^). 

Therefore, the above evidence supports a significant association between IL-6 and CAS.

### 3.7. Calcification Pathway Genes and CAS 

#### 3.7.1. Palmdelphin 

The palmdelphin gene *(PALMD)* is located on chromosome 1 at position 1p21.2. Theriault et al., in 2018 [38], initially identified *PALMD* as a gene potentially associated with CAS through transcriptomic analysis and confirmed their findings in the QUEBEC-CANS (1009 CAS, 1017 matched controls) and UK Biobank (1319 CAS and 352,191 controls) datasets. They found that rs6702619 was strongly associated with CAS in both cohorts, with a combined OR = 1.28 (CI = 1.20–1.36; *p* = 1.88 × 10^−14^). Helgadottir et al. [37] studied 2457 CAS cases from Iceland and 249,342 controls, identifying a new locus near *PALMD* (rs7543130; OR = 1.20, CI = 1.16–1.35; *p* = 1.2 × 10^−22^). Theriault et al. [19] further confirmed these findings in 2019, analysing 5115 CAS cases and 354,072 controls, and found an association for rs7543039-T with an OR = 1.20 (CI = 1.10–1.30; *p* = 1.89 × 10^−6^). Junco-Vicente et al. [37] also supported these findings, reporting that the rs6702619-G allele was associated with CAS in their study of CAS and 478 controls (OR 1.42, CI = 1.01–2.00; *p* = 0.045). Additionally, Small et al. [12], using data from the Million Veteran Program (14,451 CAS patients and 398,544 controls), found that the rs7543130 allele was associated with CAS (OR 1.14, CI = 1.11–1.17; *p* = 6.2 × 10^−24^). Furthermore, using 17 SNPs within 1 Mb of *PALMD* on two cohorts, Li et al. [39] found that this 17 SNP model explained 48% of expression variance. Both cohorts were individually associated with CAS, and when combined in a meta-analysis, the OR = 0.84 (CI, 0.80–0.88; *p* = 1.1 × 10^−12^). This is in line with the previous studies, as it investigated genetically determined higher levels of palmdelphin associating with less CAS, whilst previous SNPs like in the Theriault et al. [38] study showed decreased mRNA expression levels of PALMD in valve tissues, thus explaining the reversed OR. The effect was slightly more significant in women, as shown by Theriault [19], which may be important since aortic valves in women have more fibrosis but less calcification than men [76]. This suggests that a one-size-fits-all treatment for aortic stenosis is unlikely to be effective, and precision medicine considering the patient’s sex may be more beneficial. The study indicates that individuals with lower *PALMD* expression in the aortic valve, as predicted by their genotype, and women may benefit more from therapies that enhance *PALMD* expression.

Palmdelphin is implicated in the differentiation of cardiac progenitor cells, through an unknown mechanism. It also plays a role in cell adhesion and migration, facilitating tissue development, and can influence intracellular signalling pathways that regulate cell growth, differentiation and response to environmental changes. Understanding the genetic association, further research was conducted to uncover the mechanism. 

Han et al. [77] generated *PALMD* knockout mice using CRISPR, demonstrating that *PALMD* deficiency worsened aortic valve remodelling and impaired valve function via an NF-κB (NF-κB)-dependent mechanism. Additionally, Wang et al. [78] showed that palmdelphin might influence CAS development through the regulation of glycolysis and NF-κB-mediated inflammation.

This mechanistic understanding can enable experimental pharmacologists to develop medications that could increase palmdelphin levels or target PALMD-mediated glycolysis to reduce the incidence or progression of CAS.

#### 3.7.2. Testis Expressed 41

The *testis expressed* 41 (*TEX41*) gene is located on chromosome 2 at position 2q22. While it is predominantly expressed in testicular tissues, it may also be expressed in vascular tissues. Helgadottir et al. in 2018 [37] first identified *TEX41* as a novel gene associated with CAS. In their study involving 2457 CAS cases and 349,342 controls, they identified a variant in the *TEX41* gene, rs1830321, associated with CAS (OR = 1.20, CI = 1.12–1.28; *p* = 7.6 × 10^−8^). They further validated this by incorporating a further five studies into a meta-analysis. Investigating a total of 6886 CAS cases and 799,439 controls they confirmed an OR = 1.15 (CI = 1.11–1.20; *p* = 1.8 × 10^−13^). Similarly, Small et al. [12], in the Million Veteran Program, investigated the *TEX41* allele rs2246363 and confirmed its association with CAS (OR 1.11, CI = 1.08–1.14; *p* = 1.4 × 10^−10^). 

*TEX41* is less well characterised than other genes, and its specific role remains to be determined. It is believed to influence gene regulation and chromatin maintenance. In the context of CAS, *TEX41* may affect valvular interstitial cell behaviour or the response of endothelial cells to injury. Given that *TEX41* expression is significantly higher in men than in women, and that men have more than double the risk of CAS compared to women, *TEX41* could provide a link to this observed sex difference.

### 3.8. Endocrine Pathway Genes and CAS 

#### 3.8.1. Parathyroid Hormone

In 2009, Schmitz et al. [40] investigated the role of the parathyroid hormone *(PTH)* SNP rs6254 by comparing 538 CAS patients to 536 controls. They found that CAS patients had a higher prevalence of the *PTH* AA genotype (108 ± 20.1% vs. 71 ± 13.2%; *p* = 0.007). Similarly, Gaudreault et al. [31] studied *PTH* in 457 CAS patients and 3294 controls, finding that the same rs6254 SNP was more frequently expressed in cases than controls (0.367 vs. 0.328, *p* = 0.024). However, this result was not significant following Bonferroni correction. Theoretically, as parathyroid hormone regulates the balance of calcium and phosphate in the body, it could contribute to increased valve calcification. Currently though, there is little evidence to suggest a direct involvement of parathyroid hormone in CAS.

#### 3.8.2. Vitamin D 

In 2001, Ortlepp et al. [41] investigated the distribution of a polymorphism in the vitamin D receptor (VDR; BsmI B/b) in 100 CAS patients compared to 100 matched controls. They found that the allelic frequency of B was 35% higher in cases than in controls (*p* = 0.01). Schmitz et al. [40] also examined the role of *VDR* in their 2009 study, noting that rs4328262 was more frequent in cases (0.470 vs. 0.424) and rs2254210 less frequent (0.330 vs. 0.364). Although the *p*-values were 8.75 × 10^−3^ and 0.048, respectively, neither remained significant after Bonferroni correction.

Like parathyroid hormone, vitamin D can influence calcium metabolism and bone formation, but there is currently no strong evidence supporting their association with or causative roles in CAS. 

### 3.9. Runt-Related Transcription Factor 2 (RUNX2) and Calcium Voltage-Gated Channel Subunit Alpha1 C (CACNA1C)

Calcium signalling pathways are critical in bodily calcification processes and have been investigated in relation to CAS. Guauque-Olarte et al. in 2015 [42] investigated two cohorts, Canadian and French, with 474 and 486 CAS cases, respectively, and 2988 and 1864 controls, respectively. Two SNPs located in intron 1 of *RUNX2,* rs114193529 and rs144071310 were associated with CAS (OR = 3.49, *p* = 5.33 × 10^−5^). Additionally, this study identified a novel SNP, rs2239118, located in intron 10 of *CACNA1C,* with the G allele being 1.8 times more frequent in CAS cases compared to controls.

Mechanistically, *RUNX2* is a key transcription factor involved in osteoblast differentiation and bone formation. *CACNA1C* encodes the α-1C subunit of the L-type calcium channel, essential for calcium influx into cells. Both genes appear to influence CAS, suggesting potential targets for future research.

### 3.10. Alkaline Phosphatase, Liver/Bone/Kidney

The alkaline phosphatase, liver/bone/kidney (*ALPL*) gene, located on chromosome 1 at position 1p36.12, encodes the enzyme alkaline phosphatase (ALP), which plays a role in bone formation, liver and kidney function. ALP facilitates bone formation by hydrolysing phosphate groups from organic molecules [79], promoting the deposition of calcium and phosphate in the bone matrix. Theriault et al. [19] showed that the rs12141569-C SNP of the *ALPL* gene was associated with CAS (OR = 1.15, CI 1.07–1.23; *p* = 0.00036). Increased ALP activity in VICs can lead to higher calcium deposition in the valve tissue, contributing to CAS progression by making the aortic valve stiff and narrowed due to calcium buildup. ALPL also promotes the transformation of VICs into osteoblast-like cells, accelerating valve calcification.

### 3.11. Miscellaneous Genes and CAS

#### 3.11.1. Neurogenic Locus Notch Homolog Protein 1

Neurogenic locus notch homolog protein 1 (*NOTCH1*), located on chromosome 9 at position 9q34.3, is a key regulator of various cellular processes, including differentiation, proliferation and apoptosis. In mice, *NOTCH1* product represses the gene encoding bone morphogenetic protein 2, thereby inhibiting osteoblast-initiated calcification. Studying this effect in humans, Ducharme et al., in 2013 [43], studied a common NOTCH1 polymorphism (rs13290979) located in intron 2 in 457 CAS patients and 3294 controls. Whilst this was significantly associated with CAS (*p* = 0.003) and remained significant after correction for multiple testing, it failed to remain significant after adjusting for population stratification (*p* = 0.088). Thus, unlike in bicuspid aortic stenosis, there was no evidence to support an association between *NOTCH1* and CAS. 

#### 3.11.2. Fatty Acid Desaturase 1/2

Fatty acid desaturases 1 and 2 (*FADS1* and *FADS2*), located on chromosome 11 at positions 11q12-q13, are involved in the metabolism of polyunsaturated fatty acids (PUFA) such as omega-3 and omega-6, converting them to arachidonic and eicosapentaenoic acids. A study by Chen et al. [24] identified the rs174547 variant at the *FADS1/2* locus as being associated with CAS (OR = 0.91; CI= 0.88–0.94; *p*  =  2.5 × 10^−8^). This association was confirmed after a meta-analysis that included seven replication cohorts with a total of 312,118 individuals of whom 9395 had CAS (OR = 0.91; CI = 0.88–0.94; *p*  =  2.5 × 10^−8^). The study also showed that a higher ratio of arachidonic acid to linoleic acid was associated with CAS (OR per SD of the natural logarithm = 1.19, CI = 1.09–1.30; *p*  =  6.6 × 10^−5^), linking omega-6 fatty acid biosynthesis with CAS. Future therapeutic targets could consider addressing this metabolic pathway.

#### 3.11.3. Neuron Navigator 1

The neuron navigator 1 (*NAV1*) gene, located on chromosome 1 at position 1q32.1, encodes a protein involved in cellular electrical activity (specifically a voltage-gated sodium channel). Theriault et al. [19] included *NAV1* in their transcriptomic analysis to identify new genes associated with CAS. They found significance at SNP rs665770. In their GWAS analysis, this SNP remained associated with CAS (OR = 1.22, CI = 1.15–1.30; *p*  =  1.22 × 10^−7^). Its association with systolic and diastolic blood pressure suggests a potential hemodynamic causative link to CAS. 

## 4. Discussion

CAS is a highly prevalent condition, with its incidence expected to rise with an ageing population [80]. It is a complex disease driven by both environmental and genetic factors. While non-modifiable factors (such as sex and age) [81] and the genome (at present) cannot be altered, our growing understanding of potential genetic causative factors could help identify novel pharmacotherapeutic targets for future treatments. This systematic review highlights the extensive evidence for genetic associations with CAS, identifying specific pathways involved.

In the lipid metabolism pathway, key genes such as *LPA*, *LDL*, *PCSK9* and *APOE* are significant players in CAS. They create a pro-inflammatory and pro-calcific environment within the aortic valve itself, leading to excessive osteoblast activity in VICs and subsequent calcification. Identifying patients at risk due to elevated Lp(a) and LDL levels suggests novel therapeutic targets. Medications such as inclisiran and PCSK9 inhibitors, which lower Lp(a) and LDL, show early promise, with ongoing studies aiming to establish their efficacy in slowing CAS progression. These insights may explain why statin trials have failed despite theoretical benefits: lowering LDL alone may not suffice without addressing other factors including Lp(a) and PCSK9. Additionally, LDL may initiate valve calcification, but other mechanisms sustain the process, indicating that early intervention targeting multiple pathways could be more effective.

Moreover, the products of genes such as *IL*-6 and *IL*-10 increase CAS through inflammatory processes. *IL*-6 for instance, can activate VICs and induce their transformation into osteoblast-like cells, leading to calcium deposition [82]. Elevated IL-6 levels can disrupt the balance between matrix synthesis and degradation, promoting valve calcification and fibrosis. IL-10 can suppress pro-inflammatory cytokines and reduce oxidative stress, thereby decreasing VIC differentiation into osteoblast-like cells [83]. This may prompt a reevaluation of pharmacotherapeutic approaches, such as ACE inhibitors, which previously failed to demonstrate improved outcomes in CAS when applied broadly across all patients. Individuals with a genetic predisposition towards a heightened inflammatory state may derive the greatest benefit from these medications. Consequently, a targeted study that focuses exclusively on patients with a genetically driven inflammatory predisposition could reveal the therapeutic advantages of addressing the inflammatory pathway in this specific subgroup.

Furthermore, the calcification pathway is also implicated in the development of disease, with genes including *PALMD* and *TEX41*. *PALMD*, involved in cell adhesion and migration, has been shown to influence aortic valve remodelling and function through, for example, NF-κB-mediated inflammation [84]. Similarly to the interleukin pathways, anti-inflammatory medications targeting individuals genetically predisposed to inflammation as a result of the *PALMD* gene may warrant investigation as a potential strategy, not only for reducing the incidence but also for slowing the progression of calcific aortic stenosis. *TEX41* is also believed to affect gene regulation and chromatin maintenance whilst higher expression in men could explain the sex differences of CAS. 

Finally, genes in the endocrine pathway are involved in calcium metabolism and bone formation. Specifically, *PTH*, *VDR*, *RUNX2*, *CACNA1C* and *ALPL* play roles in CAS primarily by influencing osteoblast differentiation and calcium deposition to the valve [85,86].

It is also important to highlight that with advancing age, there appears to be a linear increase in the incidence of CAS, particularly in individuals over the age of 60. This trend suggests a potential interaction between environmental factors and genetic predisposition. Despite evidence supporting its utility, formal screening with echocardiography has not been widely implemented. A recent study demonstrated that approximately 15 echocardiograms would be needed to detect one individual over the age of 75 with any valvular disease including CAS [6]. In the future, it is plausible that genomic analysis could help identify a cohort of patients at higher risk for developing CAS, enabling more targeted screening, potential pharmacotherapy and early intervention.

This review focused on incident CAS and did not cover the genetic factors related to CAS progression. However, genes such as *LPA* have been linked to CAS progression. This is significant because, with decreasing costs in genetic and genomic testing, it may soon be feasible to offer whole genome sequencing to all CAS patients. This approach will enable clinicians to risk-stratify patients more effectively, identifying those who may experience rapid disease progression and require more frequent monitoring and early intervention, as well as those with slower progression who could have less frequent follow-ups and aid family screening.

Several challenges, however, must be addressed. Firstly, ethical considerations regarding data privacy, including which parts of the genome are reported and how incidental findings are handled, must be addressed. Secondly, infrastructure must be updated to accommodate the large amount of data generated by genomic testing. Thirdly, implementing routine genetic and genomic testing will require education and training for healthcare professionals, as well as the development of clear guidelines and protocols to guide clinicians.

Despite the challenges, targeted pharmacotherapies aim to reduce the plasma concentration of adverse lipids in the bloodstream. Identifying genetic associations opens the door to genomic medicine and genetic engineering where adverse SNPs can be modified. We are in an era of remarkable advancements in this field, with gene therapy showing transformative results in conditions such as spinal muscular atrophy [87], haemophilia B [88], Crigler–Najjar syndrome [89] and cystic fibrosis [90].

Establishing genetic associations is the crucial first step to considering gene therapy for CAS. Unlike monogenic conditions, CAS results from polygenic risk, complicating the development of effective gene therapies. Multiple pathways are involved in CAS pathogenesis, making it unlikely that modifying a single gene or SNP will reverse or slow disease progression. However, future technological advancements may enable multi-gene therapy approaches for patients with CAS, offering new hope for effective treatments. 

*Limitations*: This review includes only observational studies with inherent potential biases. Additionally, most studies included participants of European ancestry, which restricts the generalisability of the findings to other populations. The studies reviewed span nearly 30 years, with sample sizes ranging from 50 to 500,000 patients. Over this period, there have been significant advancements in genetic research and technology, convoluting direct comparisons between older and recent studies. Despite these challenges, this review represents the first comprehensive synthesis of available literature on the genetic associations of CAS.

## 5. Conclusions

CAS is highly prevalent in the older population and its incidence is projected to increase. While environmental factors are well-recognised contributors, genetic factors also play a crucial role in the development of CAS. Several genes have been found to be associated with CAS, including genes in the lipid metabolism, inflammation, endocrine and bone metabolism pathways. Understanding these genetic factors enhances our comprehension of disease mechanisms, facilitates risk-stratification for patients and their families, and has the potential to contribute to the advancement of targeted pharmacotherapies. This approach would significantly reduce the morbidity and mortality associated with CAS, ultimately improving patient outcomes.

## Figures and Tables

**Table 1:** Clinical association studies in relation to contributing pathways, presented in chronological order.

Gene(s)	Associated Metabolic Pathway	Related Publications
**LPA**	Lipid metabolism	Thanassoulis et al., 2013 [15], Arsenault et al., 2014 [16], Kamstrup et al., 2017 [17], Ozkan et al., 2019 [18], Theriault et al., 2019 [19], Trenkwalder et al., 2019 [20], Perrot et al., 2019 [21], Hoekstra et al., 2021 [22], Guertin et al., 2021 [23], Chen et al., 2020 [24], Small et al., 2023 [11]
**LDL**	Lipid metabolism	Smith et al., 2014 [25], Allara et al., 2019 [26]
**PCSK9**	Lipid metabolism	Langsted et al., 2016 [27], Perrot et al., 2020 [28]
**Apolipoprotein**	Lipid metabolism	Avakian et al., 2001 [29], Novaro et al., 2003 [30], Ortlepp et al., 2006 [31], Gaudreault et al., 2011 [32], Kritharides et al., 2017 [33], Wang et al., 2018 [34]
**Lp-PLA2**	Lipid metabolism	Perrot et al., 2020 [35]
**PONS1**	Lipid metabolism	Moura et al., 2012 [36]
**IL-6**	Inflammation	Theriault et al., 2019 [19], Junco-Vincente et al., 2022 [37], Small et al., 2023 [11]
**IL-10**	Inflammation	Gaudreault et al., 2011 [32]
**PALMD**	Calcification pathway	Helgadottir et al., 2018 [38], Theriault et al., 2018 [39], Theriault et al., 2019 [19], Li et al., 2020 [40], Junco-Vincente et al., 2022 [37], Small et al., 2023 [11]
**TEX41**	Calcification pathway	Helgadottir et al., 2018 [38], Small et al., 2023 [11]
**PTH**	Endocrine metabolism	Schmitz et al., 2009 [41], Gaudreault et al., 2011 [32]
**Vitamin D Receptor**	Endocrine metabolism	Ortlepp et al., 2001 [42], Schmitz et al., 2009 [41]
**RUNX2 and CACNA1C**	Endocrine metabolism	Guauque-Olarte et al., 2015 [43]
**ALPL**	Endocrine metabolism	Theriault et al., 2019 [19]
**NOTCH1**	Miscellaneous	Ducharme et al., 2013 [44]
**FADS1/2**	Miscellaneous	Chen et al., 2020 [24]
**NAV1**	Miscellaneous	Theriault et al., 2019 [19]

Abbreviations: ALPL= alkaline phosphatase, liver/bone/kidney; APOE = apolipoprotein E; APOB = apolipoprotein B; CACNA1C = calcium voltage-gated channel subunit alpha1 C; CAS = calcific aortic stenosis; CI = confidence interval; FADS1/2 = fatty acid desaturase ½; HR = hazard ratio; IL-6 = interleukin 6; IL-10 = interleukin 6; LDL = low density lipoprotein; Lp-PLA2 = lipoprotein-associated phospholipase A_2_; LPA = lipoprotein A; Lp(a) = lipoprotein(a); NAV1 = neuron navigator 1; NOTCH1 = neurogenic locus notch homolog protein 1; OxPL-apoB = oxidised phospholipids bound to apolipoprotein B-100; OR = odds ratio; PALMD = palmdelphin; PONS1 = paraoxonase 1; PTH = parathyroid hormone; RUNX2 = runt-related transcription factor 2; SNP = single nucleotide polymorphism; TEX41 = testis expressed 41.

**Table 2:** The risk of association of ε2 carrier status and CAS. Interaction *p* = 0.50.

	Lipoprotein(a) ≤ 50 mg/dL	Lipoprotein(a) > 50 mg/dL
ε 2 carriers	1.00	1.49 (0.72 to 3.08)
Non ε 2 carriers	1.05 (0.74 to 1.51)	2.04 (1.46 to 2.26)

## Data Availability

All data used in this study are included in the manuscript.

## Supplementary Materials

The following supporting information can be downloaded at: https://www.mdpi.com/article/10.3390/genes15101309/s1, Table S1: The PRISMA 2020 statement: an updated guideline for reporting systematic reviews; Table S2: Assessment quality and risk of bias using the Newcastle-Ottawa Quality Assessment Scale (NOS) for cohort/ observational studies; Table S3: Displaying the publication, the year, population and main findings of the studies which were utilised in this systematic review; Table S4: This table provides the detail results for each cohort and their association with calcific aortic stenosis, if they were provided in the original study.
