# Supplementary material for: Genetics of Calcific Aortic Stenosis: A Systematic Review
Source: Genes (Basel). 2024 Oct 10;15(10):1309. doi: 10.3390/genes15101309 (PMC11508093; doi:10.3390/genes15101309)
Supplement: Supplementary file 1 [file genes-15-01309-s001.zip › genes-3208720-supplementary.pdf]

Supplement for *Genetics of Calcific Aortic Stenosis: A Systematic Review*

Vassiliou et al.

Supplementary Table S1

Section and Topic	Item #	Checklist item	Location where item is reported
**TITLE**			
Title	1	Identify the report as a systematic review.	Page 1
**ABSTRACT**			
Abstract	2	See the PRISMA 2020 for Abstracts checklist.	Page 1
**INTRODUCTION**			
Rationale	3	Describe the rationale for the review in the context of existing knowledge.	Pg 2-3
Objectives	4	Provide an explicit statement of the objective(s) or question(s) the review addresses.	Pg 3
**METHODS**			
Eligibility criteria	5	Specify the inclusion and exclusion criteria for the review and how studies were grouped for the syntheses.	Pg 3
Information sources	6	Specify all databases, registers, websites, organisations, reference lists and other sources searched or consulted to identify studies. Specify the date when each source was last searched or consulted.	Pg 3
Search strategy	7	Present the full search strategies for all databases, registers and websites, including any filters and limits used.	Pg 3
Selection process	8	Specify the methods used to decide whether a study met the inclusion criteria of the review, including how many reviewers screened each record and each report retrieved, whether they worked independently, and if applicable, details of automation tools used in the process.	Pg 3
Data collection process	9	Specify the methods used to collect data from reports, including how many reviewers collected data from each report, whether they worked independently, any processes for obtaining or confirming data from study investigators, and if applicable, details of automation tools used in the process.	Pg 3
Data items	10a	List and define all outcomes for which data were sought. Specify whether all results that were compatible with each outcome domain in each study were sought (e.g. for all measures, time points, analyses), and if not, the methods used to decide which results to collect.	Pg 3
	10b	List and define all other variables for which data were sought (e.g. participant and intervention characteristics, funding sources). Describe any assumptions made about any missing or unclear information.	Pg 3
Study risk of bias assessment	11	Specify the methods used to assess risk of bias in the included studies, including details of the tool(s) used, how many reviewers assessed each study and whether they worked independently, and if applicable, details of automation tools used in the process.	Pg 3
Effect measures	12	Specify for each outcome the effect measure(s) (e.g. risk ratio, mean difference) used in the synthesis or presentation of results.	Pg 3
Synthesis methods	13a	Describe the processes used to decide which studies were eligible for each synthesis (e.g. tabulating the study intervention characteristics and comparing against the planned groups for each synthesis (item #5)).	Pg 3
	13b	Describe any methods required to prepare the data for presentation or synthesis, such as handling of missing summary statistics, or data conversions.	Pg 3

Section and Topic	Item #	Checklist item	Location where item is reported
	13c	Describe any methods used to tabulate or visually display results of individual studies and syntheses.	Pg 3
	13d	Describe any methods used to synthesize results and provide a rationale for the choice(s). If meta-analysis was performed, describe the model(s), method(s) to identify the presence and extent of statistical heterogeneity, and software package(s) used.	Pg 3
	13e	Describe any methods used to explore possible causes of heterogeneity among study results (e.g. subgroup analysis, meta-regression).	Pg 3
	13f	Describe any sensitivity analyses conducted to assess robustness of the synthesized results.	Pg 3
Reporting bias assessment	14	Describe any methods used to assess risk of bias due to missing results in a synthesis (arising from reporting biases).	Pg 3
Certainty assessment	15	Describe any methods used to assess certainty (or confidence) in the body of evidence for an outcome.	Pg 3
**RESULTS**			
Study selection	16a	Describe the results of the search and selection process, from the number of records identified in the search to the number of studies included in the review, ideally using a flow diagram.	Pg 5
	16b	Cite studies that might appear to meet the inclusion criteria, but which were excluded, and explain why they were excluded.	Pg 5
Study characteristics	17	Cite each included study and present its characteristics.	Pg 5-10
Risk of bias in studies	18	Present assessments of risk of bias for each included study.	Supplement
Results of individual studies	19	For all outcomes, present, for each study: (a) summary statistics for each group (where appropriate) and (b) an effect estimate and its precision (e.g. confidence/credible interval), ideally using structured tables or plots.	Pg 5-10
Results of syntheses	20a	For each synthesis, briefly summarise the characteristics and risk of bias among contributing studies.	Pg 11-18
	20b	Present results of all statistical syntheses conducted. If meta-analysis was done, present for each the summary estimate and its precision (e.g. confidence/credible interval) and measures of statistical heterogeneity. If comparing groups, describe the direction of the effect.	No meta-analysis done
	20c	Present results of all investigations of possible causes of heterogeneity among study results.	No meta-analysis done
	20d	Present results of all sensitivity analyses conducted to assess the robustness of the synthesized results.	No meta-analysis done
Reporting biases	21	Present assessments of risk of bias due to missing results (arising from reporting biases) for each synthesis assessed.	No meta-analysis done
Certainty of evidence	22	Present assessments of certainty (or confidence) in the body of evidence for each outcome assessed.	No meta-analysis done

Section and Topic	Item #	Checklist item	Location where item is reported
**DISCUSSION**			
Discussion	23a	Provide a general interpretation of the results in the context of other evidence.	Pg 18-20
	23b	Discuss any limitations of the evidence included in the review.	Pg 18-20
	23c	Discuss any limitations of the review processes used.	Pg 18-20
	23d	Discuss implications of the results for practice, policy, and future research.	Pg 18-20
**OTHER INFORMATION**			
Registration and protocol	24a	Provide registration information for the review, including register name and registration number, or state that the review was not registered.	Pg 3
	24b	Indicate where the review protocol can be accessed, or state that a protocol was not prepared.	Pg 3
	24c	Describe and explain any amendments to information provided at registration or in the protocol.	No ammendments
Support	25	Describe sources of financial or non-financial support for the review, and the role of the funders or sponsors in the review.	Pg 21
Competing interests	26	Declare any competing interests of review authors.	Pg 21
Availability of data, code and other materials	27	Report which of the following are publicly available and where they can be found: template data collection forms; data extracted from included studies; data used for all analyses; analytic code; any other materials used in the review.	Pg 5-10

Supplemental Table S1 indicating location in the manuscript items are reported. *From:* Page MJ, McKenzie JE, Bossuyt PM, Boutron I, Hoffmann TC, Mulrow CD, et al. The PRISMA 2020 statement: an updated guideline for reporting systematic reviews. BMJ 2021;372:n71. doi: 10.1136/bmj.n71

Supplementary Table S2

Assessment quality and risk of bias using the Newcastle-Ottawa Quality Assessment Scale (NOS) for cohort/ observational studies.

Study	Year	Selection /4	Comparability /2	Outcome /3	Total /9	Quality of study
Allara et al.	2019	***	**	***	8	Good
Arsenault et al.	2017	***	**	***	8	Good
Avakian et al.	2001	**	**	***	7	Good
Ducharme et al.	2013	***	**	***	8	Good
Chen et al.	2021	***	**	***	8	Good
Gaudreault et al.	2011	***	**	***	8	Good
Guauque-Olarte et al.	2015	***	**	***	8	Good
Guertin et al.	2021	***	**	***	8	Good
Helgadottir et al.	2018	***	**	***	8	Good
Hoekstra et al.	2021	***	**	***	8	Good
Junco-Vincente et al.	2023	**	**	***	7	Good
Kamstrup et al.	2017	***	**	***	8	Good
Kritharides et al.	2017	***	**	***	8	Good
Langsted et al.	2016	***	**	***	8	Good
Li et al.	2020	***	**	***	8	Good
Moura et al.	2012	**	**	***	7	Good
Perrot et al.	2019	***	**	***	8	Good
Perrot et al.	2020	***	**	***	8	Good
Perrot et al.	2020b	***	**	***	8	Good
Novaro et al.	2003	**	**	***	7	Good
Ortlepp et al.	2001	**	**	***	7	Good
Ortlepp et al.	2006	***	**	***	8	Good
Ozkan et al.	2019	**	**	***	7	Good
Schmitz et al.	2009	***	**	***	8	Good

Small et al.	2023	***	**	***	8	Good
Smith et al.	2014	***	**	***	8	Good
Thanassoulis et al.	2013	***	**	***	8	Good
Theriault et al.	2018	***	**	***	8	Good
Theriault et al.	2019	***	**	***	8	Good
Trenkwalder et al.	2019	***	**	***	8	Good
Wang et al.	2018	***	**	***	8	Good

Supplemental table S2 showing the results of the Newcastle Ottawa Scale for quality and risk of bias assessment. A score of 7-9 indicates high quality, 4-6 high risk of bias and 0-3 very high risk of bias. All the studies appear to be of good quality with no study showing evidence of high or very high risk of bias. Therefore, the identified results from all the studies carry significant weight.

Supplementary Table S3

Study	Year Published	Population	Main Findings
**Lipid metabolism Genes**			
**LPA**			
**Thanassoulis et al.**	2013	CAS = 6942<br>Control = 28193	After adjustment for independently- associated risk factors, single SNP rs10455872 on chromosome 6 was strongly and independently associated with incident aortic stenosis (HR per risk allele= 1.68; CI=1.32-2.15; $p=3\times10^{-5}$)
**Arsenault et al.**	2014	AS=118<br>Control=17435<br><br>Prospective Study EPIC-Norfolk<br><br>Replication Study: Montreal Heart Institute AS=309 Control=404	In EPIC-Norfolk, compared with rs10455872 AA homozygotes, carriers of 1 G allele were at increased risk of AS (HR=1.78; CI=1.11–2.87) whilst GG carriers had an even higher risk (HR=4.83 (1.77-13.20))<br><br>In the Montreal study, rs10455872 also showed a positive association with AS (odds ratio, 1.57; 95% confidence interval, 1.10–2.26)
**Kamstrup et al.**	2017	CAS = 725<br>Control= 1413	Low number of KIV-2 repeats and minor allele carrier status for rs10455872. In addition, OxPL-apoB, OxPL-apo(a), and lp(a) levels genetically determined by LPA genotype was associated with CAS OR= 1.18 (95% CI, 1.10–1.27), 1.09 (95% CI, 1.05–1.13), and 1.09 (95% CI, 1.05–1.14), respectively.
**Ozkan et al.**	2019	CAS = 75<br>Control= 77	Significantly more AA (almost x4) for rs1055872 in AS than controls (97% vs 25%); for rs3798220 all AS were TT vs 36% of controls - both values p<0.001
**Theriault et al.***	2019	AS = 5115<br>Control =354072	Study looked at the SNP rs10455872-G of the LPA gene, and found a CAS OR=1.49, CI=1.29–1.72; $9.78\times^{10-6}$
**Trenkwalder et al.**	2019	AS =4651<br>Control=8134	Presence of G allele at rs1333049 associated with CAS, OR=1.37, CI=1.27-1.47, $p=6.9\times10^{-10}$<br>Presence of C allele at rs1333049 showed a protective trend for CAS, OR=0.93, CI 0.88-0.99, p=0.014 which did not resist correction for multiple comparisons.

**Perrot et al.**	2019	AS = 9459<br>Control= 428722	Study found an association of genetically elevated LPA levels with CAVS with OR=1.49, CI=1.32-1.69, p<0.001.
**Hoekstra et al.**	2021	AS = 1722<br>Control= 291552	The most significant variant, rs10455872 in *LPA*, explained 29% of variance in Lp(a) levels. 126 other independent variants in the *LPA* region explaining a further 20% of variance.
**Guertin et al.**	2021	AS = 2574<br>Control=408403	For CAS, similar associations were observed in men and women in third tertiles divided by LPA-Weighted Genetic Risk Scores (OR =1.27, CI=1.13–1.43) in men and OR=1.22, CI, 1.04–1.44] in women).
**Chen et al.**	2021	AS =3,469<br>Control=41234	The rs174547 variant at the LPA locus was associated with AS OR=1.34, CI 1.23-1.47; p=1.5 x10^{-10}
**Small et al. ***	2023	AS = 14451<br>Control = 398544	The rs10455872 allele was associated with CAS OR=1.37, CI=1.30–1.44, p=7.76×10^{-34}
**LDL**			
**Smith et al.**	2014	AS = 473<br>Control =27988	Lipid genetic risk score for LDL was based on 57 SNPs. An LDL genetic risk score associated with CAS, HR 1.28, CI=1.04-1.57; p=0.02 adjusted for age, sex, height, weight, diabetes, hypertension, smoking<br>This could suggest that LDL associates genetically with AS and therefore reducing LDL early on may influence outcome in terms of burden of calcification and progression to more severe CAS.
**Allara et al.**	2019	AS= 2244<br>Control= 365459	Genetically predicted LDL-Cholesterol associated with CAS, OR per SD increase, 1.46 (95% CI=1.25–1.70; p<0.003)
**PCSK9**			
**Langsted et al.**	2016	AS = 1463<br>Control = 100503	Some 2606 individuals being *PCSK9* R46L carriers with 26 events vs. 100 477 noncarriers with 1437 events were

			compared; OR=0.64, CI=0.44–0.95) in analyses adjusted for sex, age, LDL cholesterol, and lipoprotein(a).
**Perrot et al.**	2020a	AS = 12059<br>Control = 541081	Association of PCSK9 R46L variant with CAS, OR=0.80, CI=0.70-0.91; p=0.001
**Apolipoprotein**			
**Avakian et al.**	2001	AS = 62<br>Control = 62	Apo ε2 carriers more common in CAS, p=0.034
**Novaro et al.**	2003	AS = 43<br>Control = 759	AS more ApoE 2/4 (9% in AS vs 2% in control) and 3/4 (28% in AS vs 23% in controls), p=0.03. In multivariable regression ApoE allele remained significant for incident AS even adjusting for age, sex, CAD, LDL , HTN and diabetes
**Ortlepp et al.**	2006	AS = 538<br>Control = 536	The frequency of the major alleles was not different between cases and controls (APOE e2: 104 (19.3%) vs. 94 (17.5%); APOE e3: 319 (59.3%) vs. 332 (61.9%); APOE e4: 115 (21.3%) vs. 110 (20.5%); all p>0.10).
**Gaudreault et al.***	2011	AS = 457<br>Control = 3297	APOB rs1042031 associated with CAS p=0.00001, SNP located 5.6kilobases downstream of the APOB stop codon rs67725189 associated p=0.000013; six SNPs around IL10 strongly associated with CAS. SNPs in PTH, TGFB1 and VDR not significant following Bonferroni correction
**Kritharides et al.**	2017	AS = 345<br>Control = 46270	ApoE ε2 carriers compared with ApoE ε2 non carriers did not have an association with AS if Lp(a) < 50. But for ε2 non carriers with Lp(a) > 50 OR=2.04, CI=1.46-2.26 with interaction p=0.50) suggesting that the effect is mediated via Lp(a) levels

**Wang et al.**	2018	AS = 314<br>Control = 652	The rs693 T allele was associated with a significantly elevated CAS risk [TT/CT vs. CC: adjusted OR = 1.58, CI= 1.82–2.10, p= 0.002; the rs6725189 T allele was also associated with a significantly elevated CAS risk (GT vs. GG: AOR = 1.82, 95% CI = 1.14–2.92, p=0.013)
**Lp-PLA2**			
**Perrot et al.**	2020b	AS =10137<br>Control=434585	Four SNPs at the PLA2G7 locus associated with either Lp-PLA2 mass or activity (rs7756935, rs1421368, rs1805017 and rs4498351). A fixed-effect meta-analysis using the inverse-variance weighted method revealed that none of the four SNPs was associated with CAVS (OR=0.99, CI 0.96 to 1.02, p=0.55). This suggests that although high Lp-PLA2 activity is linked to CAS, the genetic associations were not significant. This finding supported that Lp-PLA2 is unlikely to be a causal risk factor or therapeutic target.
**PONS1**			
**Moura et al.**	2012	AS = 67<br>Control = 251	Rs662 associated with CAS (p=0.01). Rs864580 showed no evidence of association.
**Inflammation genes**			
**IL-6**			
**Theriault et al.***	2019	AS = 5115<br>Control =354072	SNP rs2069832-A of IL-6 associated with CAS (OR 1.27, CI=1.19-1.36; $p=6.71x10^{-10}$)
**Junco-Vincente et al.***	2022	AS = 316<br>Control = 113	IL-6 rs1800795 CG associated with CAS OR=1.68, CI=1.02-2.78; p=0.043), CC OR=4.73, CI=1.61-17.90; p=0.01), C OR=1.72, CI=1.19-2.52; p=0.005. Patients with the CC allele also had higher IL-6 levels also, more than double the other alleles.

**Small et al. ***	2023	AS = 14451<br>Control = 398544	SNP rs1474347 associated with CAS, OR=1.09, CI=1.06–1.13; $p=2.27\times10^{-07}$
**IL-10**			
**Gaudreault et al.***	2011	AS = 457<br>Control = 3297	APOB rs1042031 associated with CAS p=0.00001, SNP located 5.6kilobases downstream of the APOB stop codon rs67725189 associated p=0.000013; six SNPs around IL10 strongly associated with CAS.
**Calcification pathways**			
**PALMD**			
**Helgadottir et al.***	2018	AS = 2457<br>Control = 349342	Located new AS locus, on chromosome 1p21 near PALMD rs7543130; OR=1.20, CI=1.16-1.25; $p = 1.2 \times 10^{-22}$
**Theriault et al.**	2018	AS = 1009<br>Control = 1017	rs6702619, a genotyped variant of PALMD, was the SNP most significantly associated with both CAS, OR= 1.29, CI=1.14–1.46, $P= 6.12 \times 10^{-5}$) and the expression of PALMD, $p= 5.82 \times 10^{-33}$.
**Theriault et al.***	2019	AS = 5115<br>Control =354072	Investigated the association of rs7543039-T mutation of PALMD with OR = 1.20, CI= 1.10–1.30; $p= 1.89\times10^{-6}$
**Li et al.**	2020	AS =2359<br>Control =350060	Meta-analysis of two cohorts showed predicted higher expression of PALMD inversely associated with CAS (OR per SD = 0.84, CI=0.80–0.88*;* $p = 1.1 \times 10^{-12}$). This is reversed OR because it compared predicted expression, with higher expression being the opposite of what other SNP causes.
**Junco-Vincente et al.***	2023	AS = 14451<br>Control = 398544	rs6702619 GT was associated with CAS; OR=1.37, CI=0.74-2.49; p=0.311; GG OR=1.99, CI=(1.01-3.97; p =0.049; G OR=1.42, CI=1.01-2.00; p=0.45)

**Small et al. ***	2023	AS = 14451<br>Control = 398544	rs7543130 associated with CAS OR=1.11, CI=1.08–1.15; $p=1.06\times10^{-10}$
**TEX 41**			
**Helgadottir et al.***	2018	AS = 2457<br>Control = 349342	Located new AS locus on chromosome 2q22 in TEX41 (rs1830321; OR=1.15, CI=1.11-1.20; $p= 1.8 \times 10^{-13}$).
**Small et al. ***	2023	AS = 14451<br>Control = 398544	rs2246363 associated with CAS OR=1.11, CI=1.08–1.14; $p=1.4\times10^{-10}$
**Endocrine metabolism genes**			
**PTH**			
**Schmitz et al. ***	2009	AS = 538<br>Control = 536	Patients with AS were found to have a higher prevalence of the PTH SNP rs6254 AA genotype (108±20.1% versus 71±13.2%; p = 0.007)
**Gaudreault et al.***	2011	AS = 457<br>Control = 3297	The rs6254 SNP was more frequently expressed in cases than controls (0.367 vs. 0.328, p=0.024).
**Vitamin D**			
**Ortlepp et al.**	2001	AS = 100<br>Control =100	Allelic frequency of B (rather than b) was 35% higher in cases than controls, p=0.01
**Schmitz et al. ***	2009	AS = 538<br>Control = 536	rs4328262 was more frequent in cases (0.470 vs. 0.424) and rs2254210 less frequent (0.330 vs. 0.364), no statistical significance following Bonferroni correction.
**RUNX2 and CACNA1C**			

**Guauque-Olarte et al.**	2015	AS = 960<br>Control =4852	Two cohorts investigated, a Canadian and a French one, with 474 and 486 CAS cases respectively, and 2,988 and 1,864 controls. Two SNPs located in intron 1 of RUNX2, rs114193529 and rs144071310, were associated with CAS (OR=3.49, p=5.33×10−5,). Additionally, this study identified a novel SNP, rs2239118, located in intron 10 of CACNA1C, with the G allele being 1.8 times more frequent in CAS cases compared to controls.
**ALPL**			
**Theriault et al.***	2019	AS = 5115<br>Control =354072	rs12141569-C of ALPL associated with CAS (OR=1.15; CI=1.07–1.23, p< 0.00036)
**Miscellaneous**			
**NOTCH1 Ducharme et al.**	2013	AS = 457<br>Control = 3294	rs13290979) in intron 2 of the NOTCH1 gene was significantly associated with AS (OR 1.248(1.076-1.449), p<0.0034)
**FADS1/2 Chen et al.**	2020	AS=9395<br>Controls= 312118	rs174547 variant at the FADS1/2 locus associated with CAS (OR=0.91; CI= 0.88-0.94; p = 2.5 × $^{10-8}$)
**NAV1 Theriault et al.**	2019	AS = 5115<br>Control =354072	In GWAS analysis, rs665770 associated with CAS (OR=1.22, CI=1.15–1.30; p = 1.22 × 10^{-7}).

Supplemental table S3. Table displaying the publication, the year, population and main findings of the studies which were utilised in this systematic review. A * indicates that a study reviewed multiple genes.

Supplementary table S4

Derivation cohorts	Derivation cohort size	Validation cohorts	Validation cohort size	Gene(s)	Main finding
**Guertin et al. 2021**					
UK Biobank	AS = 2574 Control= 408 403	European Prospective Investigation into Cancer and Nutrition- Norfolk	Control = 18 297<br>AS = 424	LPA	For CAS, similar associations were observed in men and women in third tertiles divided by LPA-Weighted Genetic Risk Scores (OR =1.27, CI=1.13–1.43) in men and OR=1.22, CI, 1.04–1.44] in women).
**Helgadottir et al. 2018**					
Icelandic genealogical database: deCODE genetics	AS = 2457<br>Control = 349342	1) Malmo Diet and Cancer Study<br>2) ASAP (the Advanced Study of Aortic Pathology) and Artist studies (Stockholm)<br>3) The Norwegian Nord-Trøndelag Health Study (HUNT)<br>4) UK Biobank	1) AS =470<br>Control= 15,162<br>2) AS =318<br>Control = 1,376<br>3) AS = 1,546 Control =24,235<br>4) AS = 1,844<br>Control = 406,814<br>5) AS = 251<br>Control =2,510<br>6) AS = 421<br>Control =1,634<br>Total:	PALMD	Located new AS loci, on chromosome 1p21 near PALMD rs7543130; OR=1.20, CI=1.16-1.25; p = 1.2×10^{-22}
				TEX 41	Located new AS loci on chromosome 2q22 in TEX41 (rs1830321; OR=1.15, CI=1.11-1.20; p= 1.8×10^{-13}).

		5) USA, Michigan<br>6) USA, Boston	4,850 AS cases and 451,731 and the United States	LPA	Replicated the reported association of the intronic *LPA* variant6 rs10455872 OR 1.46 (1.37–1.56), p=1.9 × 10^{-31}
**Hoekstra et al. 2021**					
UK Biobank/dbGAP	AS = 1722<br>Control= 291552	N/A	N/A	LPA	The most significant variant, rs10455872 in LPA, explained 29% of variance in Lp(a) levels. 126 other independent variants in the LPA region explaining a further 20% of variance.
				Apolipoprotein	rs1065853 in APOE was associated with decreased Lp(a) levels −0.11 [−0.12 to −0.10]; P=2.8×10-96, while rs8178824 variant in APOH was associated in an increase in Lp(a) 0.064 [0.047–0.081]; P=2.8×10-13) .
				CETP	Was associated with decreased Lp(a) levels, when variant rs247617 on chromosome 16 was identified; OR: -0.023 [−0.030 to −0.017]; P=1.0×10-13)
**Junco-Vincente et al. 2023**					
Three unnamed Spanish Cohorts	Cohort 1 = 278<br>Cohort 2 = 120<br>Cohort 3 = 336	N/A	N/A	IL6	IL-6 rs1800795 CG associated with CAS OR=1.68, CI=1.02-2.78;p=0.043), CC OR=4.73, CI=1.61-17.90; p=0.01), C OR=1.72, CI=1.19-2.52; p=0.005. Patients with the CC allele also had higher IL-6 levels also, more than double the other alleles.

				PALMD	*PALMD* rs6702619 GT/GG associated with OR 1.29 (p= 0.413) and OR 2.05(p = 0.045).
**Li et al. 2020**					
1) UK Biobank<br>2) QUEBED-CAVS	1) AS = 350393<br>Control = 1350<br>2) AS = 2026<br>Control = 1009	N/A	N/A	1) PALMD	Predicted expression in the aortic valve was inversely associated with CAVS in the UK Biobank (OR = 0.84 [0.80–0.89] per SD, $P = 9.3 \times 10^{-10}$.
				2) PALMD	Predicted expression in the aortic valve was inversely associated with CAVS in QUEBEC-CAVS (OR = 0.83 [0.76–0.91] per SD, $P = 7.4 \times 10^{-5}$)
				Meta-analysis	Meta-analysis of two cohorts showed predicted higher expression of PALMD inversely associated with CS (OR per SD = 0.84, CI=0.80–0.88;$p = 1.1 \times 10^{-12}$). This is reversed OR because it compared predicted expression, with higher expression being the opposite of what other SNP causes.
**Perrott et al. 2020a**					
1) (Copenhagen General Population Study, Copenhagen City Heart Study, and Copenhagen Ischemic Heart Disease Study,<br><br>2) UK Biobank	1) AS = 1,437<br>Control = 99,040 control patients<br><br>2) AS = 1,350<br>Control = 349,043<br><br>3) AS = 508 cases<br>Control = 20,421	N/A	N/A	Meta-Analysis of all cohorts looking at PCSK9	PCSK9 R46L variant and CAVS in 1 published and 9 previously unpublished studies totaling 12,059 CAVS cases and 541,081 control patients. Carriers of the R46L variant had lower odds of CAVS compared with noncarriers (odds ratio: 0.80 [95% confidence interval: 0.70 to 0.91]; p < 0.001).

3) EPIC-Norfolk [European Prospective Investigation into Cancer and Nutrition–Norfolk]<br><br>4)MDCS [Malmo Diet and Cancer Study]<br><br>5) GERA [Genetic Epidemiology Research on Aging],<br><br>6) The Estonian Biobank<br><br>7) QUEBEC-CAVS study<br><br>8) 3 Unnamed French cohorts	4) AS = 5,963 control =5 ,963<br><br>5) AS = 3,469 Control =41,234<br><br>6) AS = 481 Control = 7,223<br><br>7) AS = 1,009 Control =11,625<br><br>8) AS = 123 Control = 6,532				

Perrott et al. 2020b					
(IUCPQ), Québec, Canada.	AS =476<br>Control = 414	1) Quebec<br>2) UK Biobank<br>3) European Prospective Investigation into Cancer and Nutrition-Norfolk<br>4) Genetic Epidemiology Research on Aging (GERA)<br>5) Malmö Diet and Cancer Study<br>6) l'institut du thorax	1) 1009 CAVS cases and 1017 controls<br>2) 1350 CAVS cases and 349 043 controls<br>3) 504 CAVS cases and 20 307 controls<br>4) 3469 CAVS cases and 51 723 controls<br>5) 682 CAVS cases and 5963 controls<br>6) 3123 CAVS cases and 653 controls	Meta-analysis on Lp-PLA2 SNPs.	A fixed-effect meta-analysis using the inverse-variance weighted method revealed that none of the four SNPs was associated with CAVS (OR=0.99 (95% CI 0.96 to 1.02, p=0.55) for rs7756935, 0.97 (95% CI 0.93 to 1.01, p=0.11) for rs1421368, 1.00 (95% CI 1.00 to 1.01, p=0.29) for rs1805017, and 1.00 (95% CI 0.97 to 1.04, p=0.87) for rs4498351).

**Smith et al. 2023**					
The Cohorts for Heart and Aging Research in Genomic Epidemiology (CHARGE) Consortium	AS = 2245<br>Control = 4697	Malmö Diet and Cancer Study	AS = 473<br>Control =27988	LDL	Lipid genetic risk score for LDL was based on 57 SNPs. An LDL genetic risk score associated with CAS, HR 1.28, CI=1.04-1.57; p=0.02 adjusted for age, sex, height, weight, diabetes, hypertension, smoking<br>This could suggest that LDL associatesgenetically with AS and therefore reducing LDL early on may influence outcome in terms of burden of calcification and progression to more severe CAS.
**Theriault et al. 2019**					
1) QUEBEC-CAVS<br>2) (CAVS-France-1)<br>3) (CAVS-France-2)<br>4) UK Biobank	1) AS = 1009<br>Control =1017<br>2) AS = 1261<br>Control = 1305<br>3) AS = 1495<br>Control = 2707<br>4) AS = N/A<br>Control = N/A	N/A	N/A	LPA	SNP at rs10455872-G showed an OR of 1.49 (1.29–1.72), p = 9.78×10^{-6}
				PALMD	SNP at rs7543039-T showed na OR of 1.20 (1.10–1.30), p = 1.89×10^{-6}
				IL6	SNP at rs2069832-A showed an OR 1.27 (1.19–1.36), p = 6.71×10^{-10}

				ALPL	SNP at rs12141569-C showed an OR 1.15 (1.07–1.23), p = 0.00036
				NAV1	SNP at rs665770-A showed an OR 1.22 (1.15–1.30), $p = 1.22\times10^{-7}$

Supplemental table S4. In many papers multiple variants and multiple cohorts were utilized and reported. This table provides the detail results for each cohort and their association with calcific aortic stenosis, if they were provided in the original study.
